# Online positive-oriented counseling, taking vitamin D3 tablet, online lifestyle modification training on premenstrual syndrome: a 3-armed randomized clinical trial

**DOI:** 10.1038/s41598-023-43940-y

**Published:** 2023-10-03

**Authors:** Maryam Mahmoodi, Tahmineh Farajkhoda, Azadeh Nadjarzadeh, Hassan Zareei Mahmoodabadi

**Affiliations:** 1 International Campus of Shahid Sadoughi University of Medical Sciences, Yazd, Iran; 2 Reproductive Health & Clinical Psychologist, Research Center for Nursing and Midwifery Care, Non-communicable Diseases Institute, Department of Midwifery, School of Nursing and Midwifery, Shahid Sadoughi University of Medical Sciences, Yazd, Iran; 3 Department of Nutrition, School of Public Health, Shahid Sadoughi University of Medical Sciences, Yazd, Iran; 4 Department of Psychology and Educational Sciences, Yazd University, Yazd, Iran

**Keywords:** Psychology, Health care

## Abstract

Lack of absolute treatment for premenstrual syndrome (PMS), its cyclic nature, considerable prevalence (70–90%), and its mental and physical burden imply necessity of effectiveness comparison of various treatments. Although antidepressant and hormonal drugs are well-known medications for PMS, in affected women who can’t tolerate, or don’t have compatibility or compliance with these drugs, other effective treatments have always been important concern. This study aimed to compare effectiveness of online positive**-**oriented counseling, taking vitamin D3 tablet, and online lifestyle modification training on alleviating PMS. 3-armed parallel randomized clinical trial was performed on 84 20–40-year-old eligible women with PMS. 84 women were randomly (www.random.org/sequenc) allocated into three groups, but data of 77 women (1, n = 25) online positive**-**oriented counseling group (6 sessions), (2, n = 27) vitamin D3 tablet group (one vitamin D3 tablet weekly for 6 weeks), and (3, n = 25) online lifestyle training group (6 sessions) were analyzed. Vitamin D3 was measured at baseline, week6 and fallow up week10. Primary outcome variable PMS was measured with Premenstrual Symptoms Screening Tool (PSST) at baseline, week 6, and follow-up week 10. Primary outcome satisfaction with intervention method was measured using satisfaction scale at week 6 and follow-up week 10. ANOVA, Repeated Measures, and Paired samples t-test were used for statistical analysis. There was no statistically significant difference in PMS at baseline between three groups respectively (33 ± 5.8, 34.1 ± 7.1, & 35.2 ± 6.4, P = 0.500). However, at follow-up week 10, there was statistically significant difference between three groups (22.3 ± 4.3, 25.4 ± 6.5, & 31.8 ± 6.5; *P <* 0.001), with greatest improvement in online positive-oriented counseling group (*P <* 0.001). Satisfaction differed significantly among three groups at week 6 (51 ± 6.8, 46.4 ± 12, & 42.3 ± 6.3, P = 0.001) and follow-up week 10 (55.7 ± 11.6, 51.4 ± 12; & 43 ± 3.3, *P <* 0.001), with most satisfaction in positive-oriented counseling group (*P <* 0.001). All three interventions alleviated PMS, but online positive-oriented counseling was more effective and satisfying. Superiority of positive-oriented counseling implies mechanism of adaptation, better relationships, forgiveness, self-mood-regulation, and feasibility of its skills that could be continued individually by women after counseling completion. It is recommended health providers, health policymakers and managers support use of these interventions in treatment program and clinical guidelines.

Trial registration: RCT registration number: IRCT20191231045967N1, Registration date:11/02/2020, Registration timing: prospective (IRCT | Survey the effect of vitamin D3 tablet intake, positivism group consulting with changing in life style in the treatment of premenstrual syndrome in women).

## Introduction

Premenstrual syndrome (PMS) refers to a set of physical, mood, and behavioral symptoms that occur in at least two consecutive cycles in the luteal phase of menstruation and interfere with an individual, family, social, or occupational activities of the affected woman^1,2^. Considering worldwide high prevalence of PMS (70–90%), women suffering from PMS-related anger and stress require more outpatient care, must pay higher treatment costs, and have more absenteeism^3–6^.

The causes of premenstrual syndrome are not fully understood, but mood swings due to ovarian cycle activity and the effects of estradiol and progesterone on serotonin and gamma-aminobutyric acid (GABA) are major factors^7^. Low magnesium and calcium levels, vitamin and mineral deficiencies, genetic predisposition, and an unhealthy lifestyle have been suggested as possible factors^8^. Although antidepressant and hormonal drugs are well known medications for PMS, in affected women who can’t tolerate, or haven’t compatibility or compliance with hormonal or antidepressant drugs, other effective treatments has always been important concern. Numerous therapeutic interventions such as medication, surgery, supplementation (vitamins and minerals), exercise, massage, aromatherapy, yoga, light therapy, diet adjustment, herbal remedies, counseling and training, promotion of psychological capacity, cognitive-behavioral therapies, and lifestyle modification methods have been recommended, but the absolute treatment of PMS is controversial^9–12^.

Given the probability effectiveness of taking supplements to improve PMS symptoms in some studies, Iran has a higher prevalence of vitamin D deficiency than the global level. Considering a very high prevalence of 30 to 90% of vitamin D deficiency over the past two decades in countries such as China, Turkey, India, Iran, and Saudi Arabia, following the national prophylactic therapy protocol for vitamin D3 insufficiency recommended by the Ministry of Health and Medical Education of Iran^13–15^, taking vitamin D3 may reduce the risk of PMS. Various age groups in Iran have prevalence of vitamin D deficiency, according to numerous studies. Vitamin D3 as a modulator can prevent inflammation before and after menstruation and alter nerve function^16,17^. Proper intake of vitamin D3 in the diet can reduce fluctuations in sex steroid hormone or nerve function by affecting calcium levels^18^. In one study, vitamin D3 treatment was suggested as a healthy, effective, and useful treatment method to improve the quality of life of young women with severe vitamin D3 deficiency and mood disorders associated with PMS^19^. The impact of vitamin D3 consumption on some premenstrual symptoms is controversial^7,18,20,21^.

Psychological approaches can also be used for treating PMS as they reduce negative emotions during menstruation and reduce psychological symptoms. One of these approaches is cognitive-behavioral group counseling^22^. Another one is positive psychology was proposed by Martin Seligman. Seligman believes positive-oriented psychology emphasizes on dealing with the past, happiness in the present, and optimism about the future^23^. Positive-oriented psychology directly targets interpersonal relationships and educates individuals to use their abilities, boost their self-esteem, and adopt strategies to reduce negative emotions that ultimately lead to improved mood and anxiety symptoms^24^. Anxiety and depression stimulate the activity of the hypothalamic–pituitary–adrenal (HPA) axis, resulting in increased blood cortisol levels. One way to deal with stress, anxiety, and depression is to think positively. It, therefore, removes negative or destructive thoughts and emotions from the mind^25,26^. The results of a study showed that positive cognitive-behavioral therapy (P- CBT) was associated with significantly higher rates of clinically significant or reliable change for depression, negative affect, and happiness^27^. In another study, positive group counseling reduced physical symptoms, anxiety, and sensitivity in interpersonal relationships, and aggression associated with PMS, but no significant reduction was observed in depression^28^.

Another PMS-related intervention is lifestyle modification which includes diet, physical activity, self-care, non-smoking, avoiding alcohol and drug use, improving social relationships, and stress management^29–31^. Physical activity increases the levels of circulating endorphins for a short time, which acts as a temporary congestion analgesic. Furthermore, proper diet and physical activity by inducing fitness and reducing fat play a role in decreasing PMS symptoms^32^. One study confirmed an association between the type of food consumed and menstrual disorders (66.1%), and an association between physical activity and premenstrual syndrome (78.94%)^33^. Another study showed that PMS symptoms were lower in active students than in inactive individuals^30^. Besides, having the right weight and fitness is recommended to reduce PMS symptoms^31^. Pilates and aerobic exercises were shown to be involved in reducing PMS symptoms^34^.

Given the recurrent nature of PMS, affected women may experience many mental and physical changes. Finding an effective treatment for this syndrome has always been an important concern^35^. Nowadays, online psychological and educational interventions are common and have benefits such as saving time and transportation costs, the possibility of more self-disclosure in the online space, but they have some disadvantages such as fear of privacy issues or eliminating the effects of face-to-face counseling^36,37^. The reason for comparing of three different interventions in the current study was their mechanism on mood regulation through changes in neurotransmitters as main cause of PMS and comparing different kinds of interventions included phycological counseling, food supplementary and educational in one study. Hypothesis of the current study was interventions may provide treatment choices for women who don’t tolerate or haven’t compliance with anti-depressant/hormonal drugs or would like to know and use other treatments beside of anti-depressant/hormonal drugs for better control of PMS. Furthermore, it is not clear which of these interventions is more effective and creates more satisfaction in women suffering from PMS. The current study aimed to compare online positive-oriented counseling, taking vitamin D3 tablet, and online lifestyle modification training in terms of their effectiveness in alleviating PMS symptoms as well as determining the satisfaction with the intervention methods.

## Materials and methods

### Study design

This three-group parallel clinical trial was performed after receiving the code of the ethics IR.SSU.REC.1398.157 from the Ethics Committee of Shahid Sadoughi University of Medical Sciences and the registration id IRCT20191231045967N1 prospectively 11/02/2020 (IRCT | Survey the effect of vitamin D3 tablet intake, positivism group consulting with changing in life style in the treatment of premenstrual syndrome in women) of Iranian Registry of Clinical Trials. This study was conducted and reported on the basis of Consolidated Standards of Reporting Trials (CONSORT) 2010 statement. This study adheres to CONSORT guidelines.

### Sample size, sampling, and randomization

Following a similar study^38^ and taking the significance level of 5% and test power of 80%, the sample size was estimated as 75 women. However, considering an approximate 10% dropout rate, 84 women were considered as participants and were allocated in three groups.

**Figure 1:**
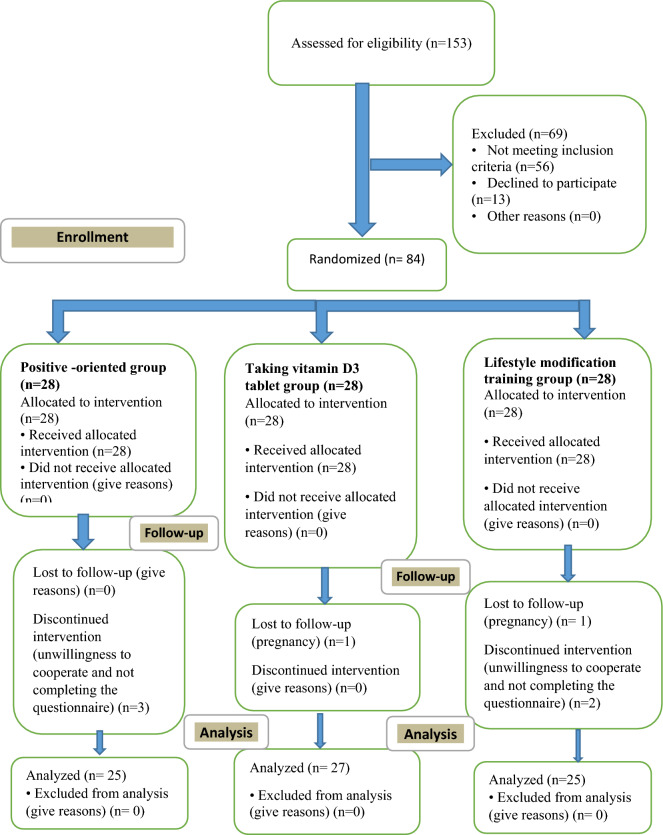
The CONSORT flow diagram.

Participants were recruited and assessed for eligibility by the first investigator among women 20–40 years old who referred to the comprehensive health centers in Arsanjan, had willingness to participation in the study, and had the self-reported PMS. 153 women who assessed for eligibility were asked to complete the PMS calendar for two consecutive menstrual cycles (to confirm of PMS with an increase of at least 30% in PMS in the second half of the menstrual cycle compared to the first half) ^1,2^. According to studies, since some diagnostic criteria for PMS is similar to PMDD (as mentioned in criterion D in Statistical Manual of Mental Disorders, DSM-5 5th edition), one of the most important common criteria to confirm PMS is use a prospective calendar for daily ratings during at least 2 consecutive symptomatic menstrual cycles to confirm the actual occurrence of PMS^1,2^. To prevent them from forgetting to complete the PMS calendar, they were sent a daily reminder text message for two months. With the outbreak of COVID-19 disease, the PMS calendars completed by women were sent to the first investigator via WhatsApp (a WhatsApp group was formed by the first investigator). 25-hydroxyvitamin D3 was measured in women who allocated into the vitamin D3 tablet group. Women with 25-hydroxyvitamin D3 serum levels less than 30 ng/ml included in the study. Out of 153 women, 69 were not eligible or not willing to continue participation in the study were excluded and 84 women who met all inclusion criteria and signed informed consent were selected as the participants. We informed the women about their right to privacy and confidentiality, their ability to withdraw at any time, their chance for allocation to each group, the details about the aims, duration and content of the interventions, and their responsibilities in the studies. Each woman received a code between 1 and 84 by the first investigator. Random allocation was done using computerized simple random numbers (www.random.org/sequenc) prepared by a biostatistician out of research team. Allocation concealment was performed using A, B, C for each group that only the biostatistician knew the name of the interventions (the first investigator and the women were not aware). The 84 sheets were prepared in same-size paper into 3 sets of 28 sheets. On one set of 28, wrote intervention A, and on the second set, wrote intervention B, and on the third wrote intervention C. These sheets were located inside the closed envelope. The selected women were randomly assigned to three groups: online positive-oriented counseling (n = 28), vitamin D3 tablet (n = 28), and online lifestyle modification training (n = 28) (Fig. 1) by the first investigator. Given a large number of women in each group and to establish better interaction with women participating in the study, the participants in each group were again simple randomly divided into two subgroups each with 14 women by a colleague out of research team. The impossibility of blinding in randomization due to the nature of the selected interventions and thus the participants were naturally aware of the type of intervention when they signed informed consent.

### Eligibility

The inclusion criteria for eligibility in the study were as follows: Women between 20 and 40 years old, living in the city of Arsanjan, having two consecutive menstrual cycles per year with at least five PMS symptoms, one of which is low mood and these symptoms begin in the second half of the menstrual cycle and improve after menstruation, experiencing at least a 30% increase in the severity of symptoms in the 5 days before menstruation according to the PMS calendar, not participating in another relevant study until the end of the current study period, having regular menstrual cycles, and having 25-hydroxyvitamin D3 level less than 30 ng/ml for vitamin D3 tablet group, not having self-reported disorders such as depression, anxiety, malnutrition, anemia, thyroid disease, diabetes, use of birth control pills, history of parathyroid surgery or parathyroid disease, not use of any herbal medicine to affect PMS symptoms, not treatment with antipsychotic drugs and other non-pharmacological methods, not participation in another relevant study until the end of the current study period. The exclusion criterion was having very sever PMS in the form of PMMD (Premenstrual Dysphoric Disorder). Withdraw reasons are stressful events, such as the death of close relatives, spousal separation, failure to attend two or more intervention sessions.

### Intervention

Women received enough explanation regarding the intervention when they singed informed consent (roles of each investigator in the study, period, date of fallow up, completion of electronic questionnaires, online joining video, and other related details). Free of charge internet was given to all three groups to participate in the online class. The online intervention was conducted in all three groups for 6 weeks (84 women with PMS) by the first investigator in collaboration with the second (principal investigator T.F., third (A.N. for taking vitamin D3 intervention), and fourth researchers (H.Z. for online positive-oriented counseling group intervention). A WhatsApp group was formed for each of the three groups (six subgroups). Women (n = 28) with 25-hydroxyvitamin D3 levels less than 30 ng/ml (blood samples of women in this group who signed informed consent measured in Arsanjan Dr. Rahim laboratory) received free of charge six vitamin D3 tablets (pearl) (50,000 IU vitamin D3, approved by FDA, made by the Zahravi factory) for six weeks (one pearl/week) recommended by the national vitamin D3 insufficiency prophylactic therapy protocol of the Ministry of Health and Medical Education of Iran^13,14^. Once a week session for six weeks was held for the vitamin D3 tablet group as reminder for taking given tablets, and assess rare side effects of vitamin D3 pearl taking, as well as alleviation of PMS. Women in vitamin D3 group, attended in Arsanjan Dr. Rahim laboratory (the only laboratory of Arsanjan for measuring 25-hydroxyvitamin D3) with compliance of COVID-19 outbreak preventive protocol. Also, for these women travel to and from the lab, and 25-hydroxyvitamin D3 measurement were for free at baseline, week 6, and follow up week 10. The first investigator M.M. attended in the lab for collecting blood sampling for all women in this group. Before signing the informed consent, the women were given sufficient and understandable explanations about of the required blood volume which obtain by syringe from arm vein, frequency of blood sampling (three times) and observance of 25-hydroxyvitamin D3 measurement conditions before test, as well as vitamin D3 rare allergic side effects and ways of its treatment by M.M (Allergic reaction was not occurred in each one of women). In addition, M.M. monitored the correctness of conducting the lab test and the accuracy of the test results. All samples were tested by ChemWell® 2910 Device by one laboratory expert. Due to low intake of vitamin D3 sources, national dress codes, and low economic status, vitamin D3 deficiency is very prevalent in Iran among all population groups (ranging from 50 to 90% according to different studies). Therefore, the Ministry of Health of Iran approved the National Guide for Vitamin D3 Supplementation Therapy (free of charge pearl 50,000 IU) for adults^13–15^. Our study participants were on the waiting for reviving vitamin D3 supplement at Arsanjan Health Center based on the national guideline of the Ministry of Health. They nearly had similar low vitamin D3 levels. However, the bio-availability and absorption of vitamin D3 through the digestive system, the parathyroid gland function, and other health conditions may vary among individuals and may cause some rare side effects such as hypervitaminosis D3 in few cases. Therefore, the ethics committee of Shahid Sadoughi University requested us to take blood samples only from the women in the group receiving vitamin D3, who were at risk of vitamin D3 side effects and possible hypervitaminosis. We followed the ethical principle of non-harm/non-maleficence in human studies and aimed to protect and provide safety for the participants and avoid unnecessary blood sampling (which is considered an invasive method). We measured vitamin D3 only in this group.

For two other groups the online positive-oriented counseling and online lifestyle modification training sessions were held in the form of online group chats, PowerPoint presentations, and video clips, and question–answer. Women were informed they can participate in online sessions with an anonymous id to protect their rights of privacy and confidentiality if they like.

The intervention for the online positive-oriented counseling group (n = 28) was conducted in groups for six weeks, each session lasting 90 min in the form of online group chats, PowerPoint presentations, and video clips, and question–answer. The strategies of sessions presented based on related studies^23,39–43^ (Table 1). Online lifestyle modification training (n = 28) was conducted for the participants in the group once a week. Each session lasted 90 min for six weeks with the content, and strategies mentioned in the literature^44^ (Table 1). In each session, the assignments done by the women and improvement in PMS were assessed for three groups. They received daily SMS reminders for doing assignments. In all three groups intervention completed after 6 weeks from baseline and fallow up was performed at week 10 from baseline. Follow up was performed by the first and the second investigators.

**Table 1:** Strategies of the intervention for the online positive-oriented counseling, and online lifestyle modification training.

Session	Content of the intervention for the online positive-oriented counseling	Content of the intervention for online lifestyle modification training
1	Looking for the positive side of life, explaining the positive psychology pyramid and its various domains in life Positive subjective experiences (savoring, happiness, gratification, fulfillment, kindness, forgiveness, and flow) in daily life	Defining physical activity, regular physical activity, adapting the physical activity and exercise program with daily life, type of exercises, and exercise during menstruation
2	Training emotion regulation skills to move from negative emotions toward positive emotions daily	Explanation about energy balance, body mass index, calories needed, recommendations for proper weight gain, physical exercises during menstruation
3	Positive institutions (families, schools, businesses, and communities) by good social relationships in daily life	Definition of nutrition, food groups, the role of food in health, nutrition during menstruation, and consumption of foods that improve premenstrual syndrome
4	Training skills to pursue optimism, hope, satisfaction, and meaning in daily life	Explanation about menstruation, hormonal changes in menstruation, mood swings before menstruation and ways to deal with them, the importance of menstrual health and prevention of premenstrual symptoms by taking adequate rest, meditation, and stress management
5	Seeking for mental growth and positive development such as creativity	Education about effects of tobacco, and substance abuse, smoking and diseases, smoking and its effect on menstruation and premenstrual syndrome in women
6	Expressing the impact of positive thinking and optimism instead of negative thoughts and rumination on the women’s happiness and well-being especially due to premenstrual syndrome, and summing up the content of the sessions	Explanation about principles of self-care for early prevention and diagnosis, assessment of healthy lifestyle skills, and summing up the content of the sessions

### Instruments and outcome variables

All study questionnaires were completed by women through electronic link via WhatsApp with giving necessary explanation. The following instruments were used to collect the data:

***1. The demographic information form:*** The form was filled in baseline to determine of age, education, awareness of having PMS, and the half of the menstrual cycle.

***2. Premenstrual Symptoms Screening Tool (PSST):*** The tool contains 19 items, of which 14 items assess mood, physical, and behavioral symptoms, and 5 items measure the effect of these symptoms on people's lives. Each item is scored from 0 to 3^45^. Cronbach's alpha value was 0.9, confirming the internal consistency of the instrument.

**Primary outcome variable:** PSST was used for measurement of PMS as the primary outcome variable in baseline, week 6, and follow-up at week 10.

***3. The Intervention Satisfaction Scale:*** This researcher-made scale was developed based on related studies in the literature^46,47^ (Cronbach's alpha of 0.87) and the items are scored on a numerical scale ranging from 1 to 10.

**Primary outcome variable:** The scale was completed by the participants in the three groups to measure the primary outcome, i.e., satisfaction with the intervention method at week 6 and week 10.

***4. Measurement of vitamin D3 serum amount:*** After separating blood serum using the ChemWell® 2910 Device (Awarance Co., USA), All blood samples were centrifuged in Arsanjan Dr. Rahim laboratory for 2 min at 300 Revolutions Per Minute (RPM) and were measured by the enzyme-linked immunosorbent assay (ELISA) method. The measurement accuracy was 0.04 and to reduce the error before placing the samples, the device was calibrated and during the measurement process, the curves were checked for each test separately. Vitamin D3 was measured at the baseline, week 6, and follow-up at week 10 for all women in the vitamin D3 tablet group.

**Secondary outcome variable:** Vitamin D3 was measured at the baseline, week 6, and follow-up at week 10 for all women in the vitamin D3 tablet group.

### Statistical analysis

The participants’ demographic data analyzed using ANOVA, Chi-square, and Fisher's exact test. Shapiro–Wilk test confirmed the normality of data distribution in the three groups. Thus, parametric tests were used to assess the research variables. The main primary outcome variable of PMS in within groups comparison was assessed by Repeated Measures, and in between groups comparison measured by ANOVA. Within groups comparison was assessed by Paired samples t-test, and between groups comparison measured by ANOVA to evaluate of another main outcome variable of satisfaction with the intervention method. Secondary outcome variable vitamin D3 was measured via Repeated Measures for comparison of 25 hydroxy-vitamin D3 serum levels at baseline, week 6, and week10. All statistical calculations were performed at the significance level of less than 0.05 (*P <* 0.05). Data were analyzed by a statistician out of the research team.

### Ethical approval and consent to participate

The Ethics Committee of Shahid Sadoughi University of Medical Sciences and the registration id IRCT20191231045967N1 prospectively11/02/2020 of Iranian Registry of Clinical Trials (IRCT | Survey the effect of vitamin D3 tablet intake, positivism group consulting with changing in life style in the treatment of premenstrual syndrome in women) approved study protocol (IR.SSU.REC.1398.157). We followed all the ethical principles of the World Medical Association Declaration of Helsinki for medical research involving human subjects. We obtained oral and written informed consent from all study participants before their recruitment.

## Results

Of 84 women participated in the study, seven women withdrew the study included three in the online positive-oriented counseling group, one in taking vitamin D3 group, and three in the online positive-oriented counseling group (Reasons were presented in consort follow chart 1). The data of 77 women were analyzed. Data were analyzed by a statistician out of the research team. The three groups did not differ significantly in mean score age, education, awareness of having PMS, and the half of menstrual cycle at the baseline (Table 2).

**Table 2:** Comparison of the participants’ demographic data in the three groups.

Variable	Positive-oriented counseling group(n = 25)	Taking vitamin D3 tablet group(n = 27)	Online lifestyle modification training (n = 25)	*P*-value
Age (years)	**Mean ± Sd**	**Mean ± Sd**	**Mean ± Sd**	
	33.75 ± 4.16	31.93 ± 5.59	33.04 ± 3.61	0.380
	**Percent (Frequency)**	**Percent (Frequency)**	**Percent (Frequency)**	
Education				
High school	16% (4)	22.2% (6)	20% (5)	0.110
Diploma	24% (6)	37% (10)	56% (14)	
Bachelor	40% (10)	37.5% (10)	20% (5)	
Masters' degree and higher	20% (5)	3.7% (1)	4% (1)	
Awareness of having PMS				
Yes	48% (12)	29.6% (8)	40% (10)	0.540
No	52% (13)	70.4(19)	60% (15)	
Half of menstrual cycle at the baseline				
First half	44% (11)	55.6% (15)	40% (10)	0.500
Second half	56% (14)	44.4% (12)	60% (15)	

ANOVA for comparison of age in three groups.

Chi-square for comparison of awareness of having PMS, and half of menstrual cycle at the baseline in three groups.

Fisher's exact test for comparison of education in three groups.

Comparison amount of Vitamin D3 at baseline, week 6, and week10 was demonstrated in Table 3 with statistically significant difference (*P <* 0.001) as secondary outcome variable.

**Table 3:** Comparison of 25 hydroxy-vitamin D3 serum level at baseline, week6, and week10.

Vitamin D3(n = 27)ng/ml	
Baseline	16.92 ± 4.13
week 6	33.32 ± 9.20
week 10	18.05 ± 5.10
P-value	< 0.001

Repeated Measures for comparison of 25 hydroxy-vitamin D3 serum level at baseline, week6, and week10.

There was no statistically significant difference in terms of PMS in the baseline between the three groups respectively (33 ± 5.8, 34.10 ± 7.10, & 35.20 ± 6.40, P = 0.500). However, at week 10 follow-up, there was a statistically significant difference (*P <* 0.001) between the three groups (22.3 ± 4.3, 25.4 ± 6.5, & 31.8 ± 6.5; *P <* 0.001), with the greatest improvement, was observed in the online positive-oriented counseling group (Table 4).

**Table 4:** Comparison of mean PMS scores in the three groups.

Premenstrual syndrome	Positive-oriented counselling group (n = 25) Mean ± SD	Vitamin D3 tablet group (n = 27) Mean ± SD	Lifestyle modification training group (n = 25) Mean ± SD	*P*-value	*P*-value
Baseline	33 ± 5.8	34.1 ± 7.1	35.2 ± 6.4	0.500	< 0.001
Week 6	26.7 ± 5.9	29 ± 5.1	32.3 ± 6.2	0.004	
Week 10	22.3 ± 4.3	25.4 ± 6.5	31.8 ± 6.5	< 0.001	
*P*-value	< 0.001	< 0.001	0.021		

ANOVA for comparison of main primary outcome premenstrual syndrome mean scores between groups.

Repeated measures for main primary outcome comparison of premenstrual syndrome mean scores within groups.

The satisfaction with the intervention method differed significantly among the three groups at week 6 (51 ± 6.8, 46.4 ± 12, & 42.3 ± 6.3, P = 0.001) and follow-up at week 10 (55.7 ± 11.6, 51.4 ± 12; & 43 ± 3.3, *P <* 0.001), with the highest mean score of satisfaction in the positive-oriented counseling group (*P <* 0.001) (Table 5).

**Table 5:** Comparison of mean satisfaction with the intervention score in the three groups.

Satisfaction with the intervention method	Positive-oriented counselling group (n = 25) Mean ± SD	Vitamin D3 tablet group (n = 27) Mean ± SD	Lifestyle modification training group (n = 25) Mean ± SD	*P*-value	*P*-value
Week 6	51.0 ± 6.8	46.4 ± 12.0	42.3 ± 6.3	0.001	0.001
Weeks 10	55.7 ± 11.6	51.4 ± 12.0	43.0 ± 3.3	< 0.001	
P-value	0.45	0.04	0.622		

ANOVA for comparison of main primary outcome satisfaction with the intervention method mean scores between groups.

Paired samples t-test for main primary outcome comparison of satisfaction with the intervention method mean scores within groups.

## Discussion

The results of the study suggested that all three interventions caused a statistically significant decrease in the PMS scores as primary outcome at week 10 compared to the baseline. Positive-oriented counseling had a greater effect on alleviation of PMS than the other two interventions. A review of the literature found no similar study addressing the same interventions examined in the current study. Thus, this section compares the results of the present study with the findings of similar studies. The Results of a study showed that cognitive-behavioral therapy (CBT) mixed with calcium and vitamin D3 pills significantly reduced PMS^48^. CBT can improve health-related quality of life in students with PMS^49^. Findings of a research showed that group stress management counseling was effective in reducing PMS symptoms^12^. Another research showed that three sessions of self-awareness counseling play an important role in reducing the PMS severity^50^. Internet-based cognitive-behavioral therapy (ICBT) can reduce the symptom severity of PMS while improving perimenstrual quality of life^51^. Although the counseling interventions in these studies differed from the counseling techniques used in the current study, it seems that counseling can empower a person to solve problems by making positive cognitive changes from negative thoughts to positive thoughts and enhancing coping skills. Accordingly, one of the reasons for the positive effect of positive counseling is its direct impact on interpersonal relationships and empowerment of people to use their abilities, and reinforcement of self-confidence and positive thinking, followed by the elimination of destructive and negative thoughts to improve their mood^26,43^. The results of the above studies were consistent with the findings of the present study.

Vitamin D3 level was increased in week 6 and decreased in week 10 in comparison with baseline in current study. A review study showed that taking calcium and vitamin D3 tablet or following a diet rich in these two substances can restore serum levels and eliminate or reduce the PMS symptoms. Calcium and vitamin D3 supplementation are recommended as a cheap, low-risk, acceptable, and available method to reduce the PMS ^52^. The results of a study showed that the vitamin D3 tablet improved the PMS significantly^53^. Findings of another study showed no relationship between PMS and serum levels of vitamin D, which was not consistent with the results of the present study confirming the relationship between decreasing mean scores of PMS and increased serum level of 25-hydroxyvitamin D3. Since vitamin D3 deficiency and insufficiency are prevalent in Iranian women, our finding seems reasonable, because after 6 weeks treatment with vitamin D3 tablet (25-hydroxyvitamin D3 reached to normal level), PMS was decreased, and between week 6 to week 10 that women did not take vitamin D3 tablet (25-hydroxyvitamin D3 decreased from its normal level), PMS was increased. Although Rajaei et al. (2016)^16^ used the same screening tool for PMS (PSST) and measurement of 25-hydroxy-vitamin D3 by ELISA kit as the current study, but their results were different. This could be because they did not use the PMS calendar to confirm PMS for two consecutive cycles and might have overestimated the PMS diagnosis in their study. In the current study the average 25-hydroxyvitamin D3 in the baseline was 16.928 ng/ml. At week 6 with weekly taking consumption of vitamin D3 tablets, its average reached 33.3 ng/ml (at normal level), and associated with a decrease in PMS scores. However, since at 6 to 10 weeks, when vitamin D3 intake was eliminated due to normalization of vitamin D3, the average vitamin D3 in this group decreased again to 18,059, leading to vitamin D3 deficiency and less improvement in PMS. It should be noticed that dose and duration of vitamin D3 intake, the inclusion and exclusion criteria, measurement techniques and intervals, and the calibration of the instruments varied in different studies. In the present study, the improvement in PMS appeared to be dependent on the serum levels of vitamin D3.

The results of the current study indicated although lifestyle modification training intervention led to a significant reduction in PMS, this reduction was less than the other two interventions. In a study, the web-based lifestyle training could relief PMS in university students^54^. Findings of another study showed that adolescents with PMS with a high-quality diet reported less depression, anxiety or change in sleep patents than those with low-quality diet^55^. The Results of a research showed a significant relationship between the severity of PMS and lifestyle factors such as eating habits, sleep patterns, stress management, and exercise^56^, confirming the findings of the present study. The reason for the less decrease in PMS symptoms compared to positive-oriented counseling can be traced to the behavioral nature of lifestyle changes, because behavioral changes usually require a longer period to show their effects^57^. Assessment the relationship between lifestyle factors and PMS showed that physical activity, inactive tobacco exposure, and frequent use of sweet-tasting foods, high consumption of fast foods, reduced consumption of vegetables and fruits, and increased caffeinated beverages explain only 52% of the changes in PMS^32^.

General reason for effectiveness of three interventions in current study may relate to unawareness of most of women in about PMS and they had not received any treatment before participating in the study. Thus, it seems that all three interventions were effective in informing them about their disease^58–60^. Moreover, online intervention might have been interesting for the women making them pay more attention to the content of the interventions. However, the greater effect of positive-oriented counseling than the other two can be attributed to the cognitive mechanism of the positive-oriented approach, which is easy to perform and does not require special preparations or leaving the house, which can be easily performed at home especially during the COVID-19 outbreak. Another reason may relate to feasibility of skills and assignments that could be continued individually by women after completion of the counseling at week 6. It seems that women had enough time to repeat learned skills individually and obtained more talent. It also seems that positive thinking and positive self-talking with regular practice over time maintain its lasting effect in the mind and increase adaptation and self-regulation, improve relationships with others, forgiveness, and physiologically increase mood-regulating neurotransmitters such as serotonin and endorphins in the body and thus improving mood and thus premenstrual syndrome^40,58,59^.

Another primary outcome variable of this study was satisfaction with the intervention method with considerable score of satisfaction in three groups. This finding would be due to short-term intervention that women could feel quick improvement in PMS after 6 weeks. In addition, understandability, acceptability and feasibility of intervention, and popularity of online intervention may be other reasons. The satisfaction with the intervention method differed significantly among the three groups with the most satisfaction in the positive-oriented counseling group and the least satisfaction in the lifestyle modification training group. This can be attributed to healthy lifestyle exercises such as dietary regimens, avoiding stressors, getting enough sleep and rest, which seems to be not easily possible and requires more willpower, practice, and perseverance compared to the two other intervention methods^61^.

Considerable satisfaction with the intervention method (this variable not assessed in other studies), women’s PMS approval using PMS calendar completion prospectively for two consecutive menstrual cycles before initiation of the intervention (it have not been reported in most of similar studies), participation of women with 25-hydroxyvitamin D3 levels less than 30 ng/ml through laboratory measurement before taking vitamin D3 pearl, one month free of intervention follow up after trial completion for reassurance of interventions effectiveness (less mentioned in similar studies), providing content for sessions as educational package for other investigators, and effectiveness of three various short-term interventions may imply strengths of the current study and its clinical application through provide treatment options for women who prefer not to use anti-depressant/hormonal drugs for their side effects or compliance , or would like to use other treatments beside of anti-depressant/ hormonal drugs for more effectiveness on PMS. The authors recommend investigating the impact of such interventions with longer term follow up in the future studies. The impossibility of blinding in intervention due to the nature of the selected interventions and thus the participants were naturally aware of the type of intervention was considered as study limitation. Another study limitation was need to internet literacy for online interventions (except taking vitamin D3 group) that is not applicable for illiterate women.

The three non-antidepressant/non-hormonal drugs interventions online positive-oriented counseling, vitamin D3 tablet, and online lifestyle modification training alleviated PMS symptoms, but the positive oriented counseling was more effective than the other two interventions. The women were also satisfied with the allocated intervention method, but more satisfaction observed in positive-oriented counseling group. Superiority of online positive-oriented counseling may imply feasibility of its skills that could be continued individually by women after termination of the counseling, and without leaving the house. It is recommended that health care providers, policymakers, and managers should support and establish interventions for women with PMS in their treatment program and clinical guidelines.

## Data Availability

The datasets used and/or analyzed during the present study are available from the corresponding author on reasonable request.

## Abbreviations

PMS: Premenstrual syndrome
ICBT: Internet-based cognitive-behavioral therapy
DRSP: Daily record of severity of problems
PMDD: Premenstrual dysphoric disorder
PSST: Premenstrual symptoms screening tool
RPM: Revolutions per minute
ELISA: Enzyme-linked immunosorbent assay
GABA: Gamma-aminobutyric acid
IRCT: Iranian registry of clinical trials
COVID: Coronavirus disease
HPA: Hypothalamic–pituitary–adrenal
PMMD: Premenstrual dysphoric disorder
ID: Identification
